# Two new *Phlegmariurus* species (Lycopodiaceae) and one new combination from Peninsular Malaysia

**DOI:** 10.3897/phytokeys.96.20878

**Published:** 2018-04-17

**Authors:** Ruth Kiew, Imin Kamin

**Affiliations:** 1 Forest Research Institute Malaysia, 52109 Kepong, Selangor, Malaysia

**Keywords:** *Phlegmariurus*, limestone, montane forest, Lycopodiaceae, new species, new combination, Peninsular Malaysia

## Abstract

Two new species, *Phlegmariurus iminii* Kiew (Lycopodiaceae) from limestone karst and *P. monticola* Kiew from montane habitats, are described from Peninsular Malaysia and a new combination is made for *Phlegmariurus pinifolius* (Trevis.) Kiew. *Phlegmariurus iminii*, known from a single hill threatened by quarrying, is Critically Endangered; while *P. monticola* and *P. pinifolius* that are relatively widespread are of Least Concern.

## Introduction

The last published flora account of Lycopodiaceae for Peninsular Malaysian with keys and species descriptions was that of Ridley (1919) who recorded 13 species. The most recent reliable checklist (Parris and Latiff 1997) listed 12 species. At the generic level, molecular studies show that *Lycopodium* L. *s.l.* is paraphyletic (Wikström and Kenrick 2001; Field et al. 2016) and should be divided into 16 monophyletic genera (PPG I 2016) of which *Diphasiastrum* Holub, *Huperzia* Bernh., *Lycopodiastrum* Holub, *Lycopodium* L., *Palhinhaea* Franco & Vasc., *Pseudodiphasium* Holub and *Pseudolycopodiella* Holub are each represented by a single species in Peninsular Malaysia and *Phlegmariurus* Holub, which is more diverse, has 12 species including the two new species described below, bringing the total for the family to 19 species.

Revision of the family currently underway for the Flora of Peninsular Malaysia and the focus of botanical exploration by the Flora of Peninsular Malaysia team (Kiew and Rafidah 2007) have brought to light two new species of *Phlegmariurus*, one an epiphyte on trees growing on limestone; the other an epiphyte on trees in lower to upper montane forest.

## Materials and methods

Specimens of all Peninsular Malaysian *Phlegmariurus* species and those of the surrounding region (Thailand, Sumatra and Borneo) in the herbaria at BM, K, KEP, KLU and UKMB (acronyms follow Thiers et al. 2017, continuously updated) were examined. Type material was examined in these herbaria as were type images relevant to this study available on JSTOR Global Plants website (http://jstor.org). Literature relevant to the region (West Malesia and Thailand), including protocols, were consulted. Conventional methods employed in herbarium taxonomy were applied in this study. All measurements were taken from dried herbarium specimens. Photographic documentation was taken from living specimens in their natural habitat. Provisional conservation assessments follow the guidelines in IUCN (2012) and Chua (2010).

## Taxonomic account

### Phlegmariurus iminii

Taxon classificationPlantaeLycopodialesLycopodiaceae

Kiew sp. nov.

urn:lsid:ipni.org:names:77177909-1

Figures 1 , 2

#### Type.

Malaysia. Pahang, Merapoh District, Gua Gunting. 30 May 2013, Imin et al. FRI 78296 (holotype KEP! barcode 235330; isotype SING!).

#### Description.

Medium-sized, tufted epiphyte. **Stems** lax, pendulous, ca. 30 cm long, slender, terete, ca. 2.5–3 mm in diameter; branching dichotomously 4 times, branches equal. **Leaves** spaced 3–4 mm apart, arranged in two alternating subspiral whorls of three, ascending at ca. 40° to stem, sessile; lamina dark green, thin and papery, lanceolate, (7–)10 × 1.8–2 mm, base flat, cuneate, margin flat, entire, narrowed to a minutely apiculate apex, glabrous above and beneath; midrib distinct on both surfaces, prominent above, keeled beneath. **Strobilus** slender ca. 1 mm thick, branched once dichotomously near the base with a short stalk 8–14 mm long and branches ca. 20 mm long followed by a short 50–90 mm long section of stem with sterile leaves, terminating in a strobilus (20–)40–70 mm long. **Sporophylls** distinct from leaves, spaced along the axis, sessile, ovate, much smaller than leaves, 1.5–2 × 0.75–1 mm, keeled on outer surface, base rounded and concave around the sporangium, margin entire, apex acute. **Sporangium** broadly reniform, ca. 2 mm long and wide, creamy becoming yellow when mature. **Spores** isotetrahedral with convex margins, polar axis ca. 20 µm, distal surface minutely foveolate.

**Figure 1.**
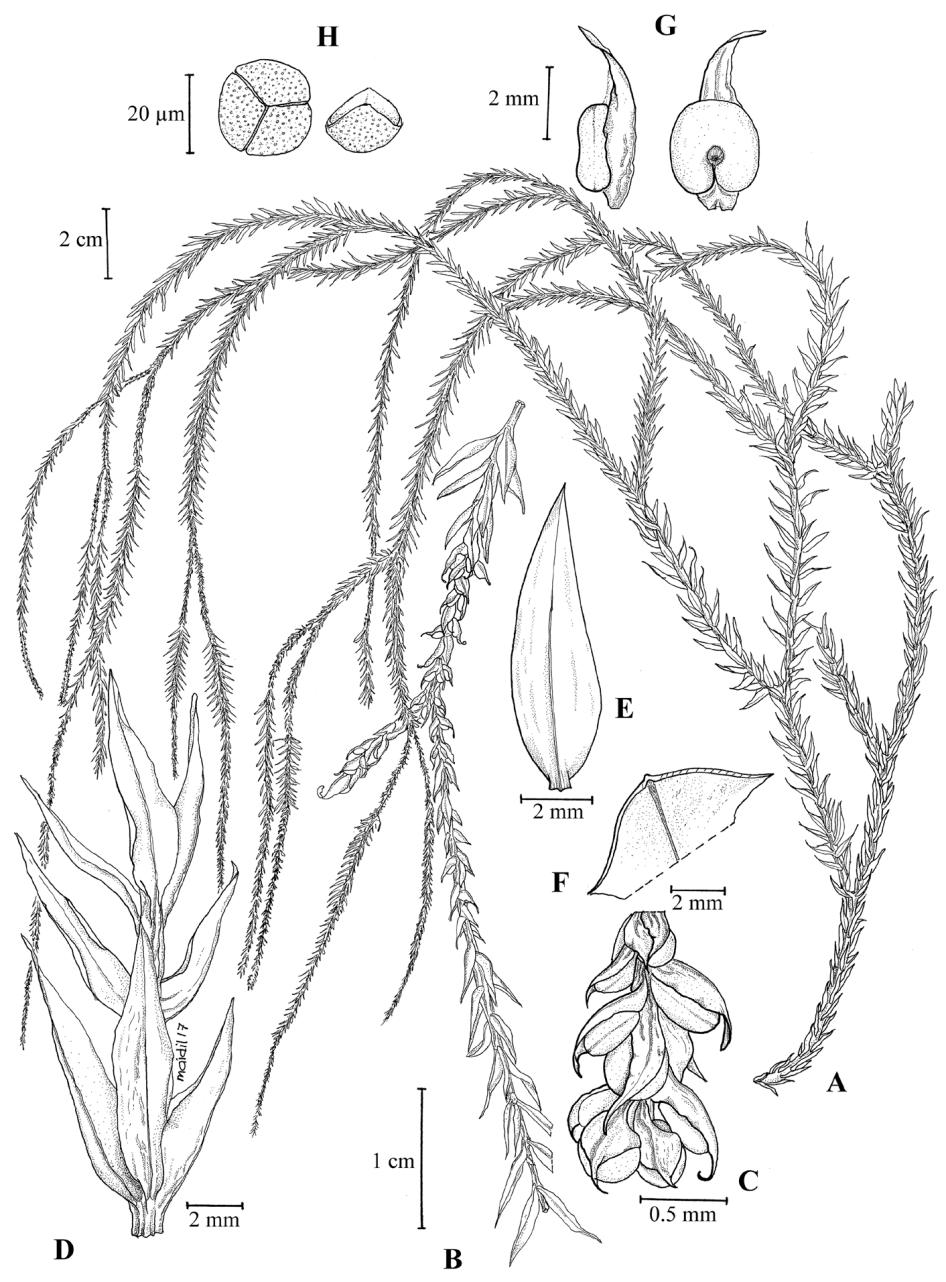
*Phlegmariurus iminii*. **A** habit **B** strobilus **C** tip of strobilus with sporangia and sporophylls **D** portion of leafy stem **E** distinct midrib on upper leaf surface **F** section of leaf to show keel **G** top view of sporangium and sporophyll **H** spores. (Drawing by Mohamad Aidil Noordin from Imin et al. FRI 81470).

**Figure 2.**
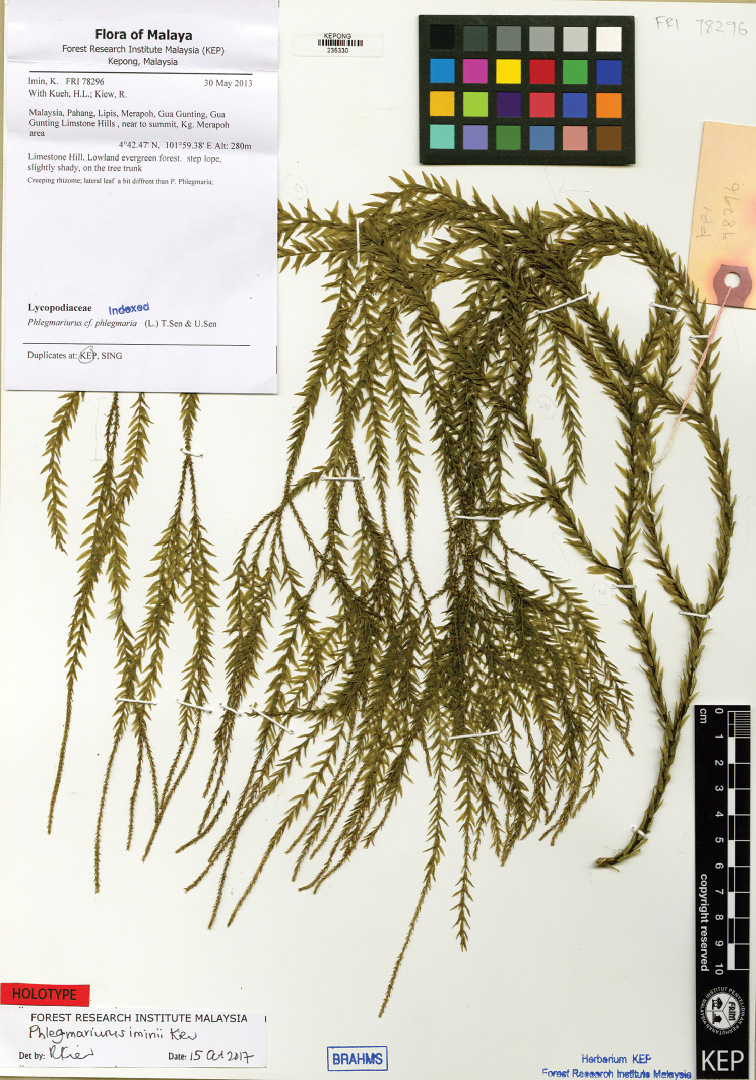
Holotype specimen (*Imin et al. FRI 78296*) of *Phlegmariurus iminii* Kiew.

#### Diagnosis.

This new species belongs to the *Phlegmariurus phlegmaria* group of species in being a medium-sized epiphyte with pendent stems, flat leaves broadest at base with a distinct midrib and sporophylls much smaller than vegetative leaves. Amongst Peninsular Malaysian species, it is different from *P. phlegmaria* (L.) T.Sen & U.Sen and *P. salvinioides* in its narrower, sessile leaves, 7–9 × 1.8–2 mm, positioned at 40° to the stem (vs. broader, subpetiolate leaves 5–15 × 2–5.5 mm, perpendicular to the stem) and in its unbranched strobili (vs. much branched). While the distinction between *P. phlegmaria* and *P. salvinioides* (Herter) Ching tends to intergrade, *P. iminii* is distinct without any specimens of these two species resembling it. It is similar to *P. pinifolius* (Trevis.) Kiew in its narrow, sessile leaves but its leaves are less crowded and positioned at 40° to the stem (vs. crowded and perpendicular to the stem) and its strobili are short and unbranched (vs. long and branched from the base). It also superficially resembles *P. banayanicus* (Herter) A.R. Field & Bostok from the Philippines but that species has broader leaves narrowed to the base (usually 8–10 × 2–3 mm) that are perpendicular to the stem and has branched inflorescences. *Phlegmariurus iminii* is distinct from all these species by a combination of its narrow, sessile leaves positioned at 40° to the stem and its straight, short, unbranched strobili with sporophylls that are not strongly appressed to the stem (Table 1).

**Table 1.** *Phlegmariurus iminii*, *P. banayanicus*, *P. pinifolius*, *P. phlegmaria* and *P. salvinioides* compared.

Character	*iminii*	*banayanicus*	*pinifolius*	*phlegmaria*	*salvinioides*
Leaf shape	lanceolate	lanceolate	elongate-lanceolate to narrowly lanceolate	ovate-lanceolate to lanceolate	lanceolate
Leaf base	cuneate	rounded	slightly narrowed	rounded to cordate	rounded to broadly truncate
Leaf angle to stem	45°	90°	90°	90°	90°
Leaf apex	narrowed to attenuate	acuminate	extremely acuminate	acute	acute
Leaf size (mm)	7–9 × 1.8–2	8–12 × (1–)2–3	6–8 × 1–1.7	4–20 × 2.5–6	5–9.5 × 2–5
Strobili	dichotomous branch at base, branches and straight	branched 2-3 times, branches curving	2, 4 or 6 branches, branches straight	much branched, branches curving	much branched, branches straight
Sporophylls	not appressed	appressed	appressed	appressed	not appressed
Leaf attachment	sessile	subpetiolate	sessile	subpetiolate	subpetiolate
Leaf spacing	spaced	spaced	very compact	spaced	spaced

#### Distribution.

In Peninsular Malaysia, it is at present known only from the type locality, Pahang, Merapoh District, Gua Gunting. Ashley R. Field (pers. comm.) notes that the species is a target for plant collectors and it is in cultivation from parts of Thailand and other parts of Peninsular Malaysia from a variety of habitats, although we have not been able to locate it in commercial nurseries in Malaysia.

#### Provisional conservation status.

Critically Endangered (A1d, B2ab[iii,v]). Its only confirmed locality is a single karst limestone hill that lies outside the network of Totally Protected Areas and has been threatened by quarrying for cement and is surrounded by oil palm plantations that expose it to disturbance from agricultural activities, in particular by the practice of clearing vegetation by burning. The limestone flora occupies only 0.4% of land area but is biodiverse harbouring at least 14% of the Peninsula’s vascular flora (Chin 1977), so it is particularly vulnerable to disturbance (Kiew 1997). In addition, many of its species like *Phlegmariurus iminii* are known from less than five limestone hills (Kiew et al. 2017).

#### Ecology.

The type specimen grew on a tree on a steep slope, slightly shaded near the summit of a limestone karst hill.

#### Etymology.

Named after Imin Kamin, Research Assistant In-Charge of the lycophyte and fern collection in the Forest Research Institute Malaysia Herbarium (KEP), who discovered this species.

### Phlegmariurus monticola

Taxon classificationPlantaeLycopodialesLycopodiaceae

Kiew sp. nov.

urn:lsid:ipni.org:names:77177910-1

Figures 3 , 4A

#### Type.

Malaysia. Pahang, Cameron Highlands, Gunung Beremban, trail from the Parit Falls to MARDI. 24 May 2007, Nor Ezzawanis & Zamri FRI 54517 (holotype KEP! barcode KEP139948; isotype KEP! barcode KEP139947).

#### Description.

Small epiphyte, tufted with (1–)6–7 stems. **Stems** pendulous or semi-erect, 11–22(–32.5) cm long, 1–2 mm diam., 1.2–1.7 cm wide across the leaves, green, branched once or sometimes twice towards the apex. **Leaves** crowded, spreading more-or-less at right angles to the stem, more-or-less in 6 rows, mid-green, soft, sessile, narrowly lanceolate to subulate, 8–10 × 0.75–1.5 mm, acutely narrowed to a sharp point at apex, margin entire, minutely revolute, glabrous above and beneath, midrib obscure above, prominent beneath. **Strobilus** green or yellowish-green, in pairs, each branching dichotomously once or twice, sometimes unbranched, (7.5–)11–12(–22) cm long, ca. 3 mm diameter. **Sporophylls** similar in shape to leaves but smaller and more compact, transition to strobilus gradual. Sporophylls sessile, ascending, arranged in 4 rows, narrowly lanceolate, 3.5–5(–7) × 0.5–1 mm at base, abruptly narrowed above the sporangium and 0.25 mm wide, margin entire. Sporangium yellow, broadly reniform, ca. 1–1.5 mm across. **Spores** isotetrahedral with convex margins, polar axis ca. 20 µm, distal surface minutely fossulate-foveolate.

**Figure 3.**
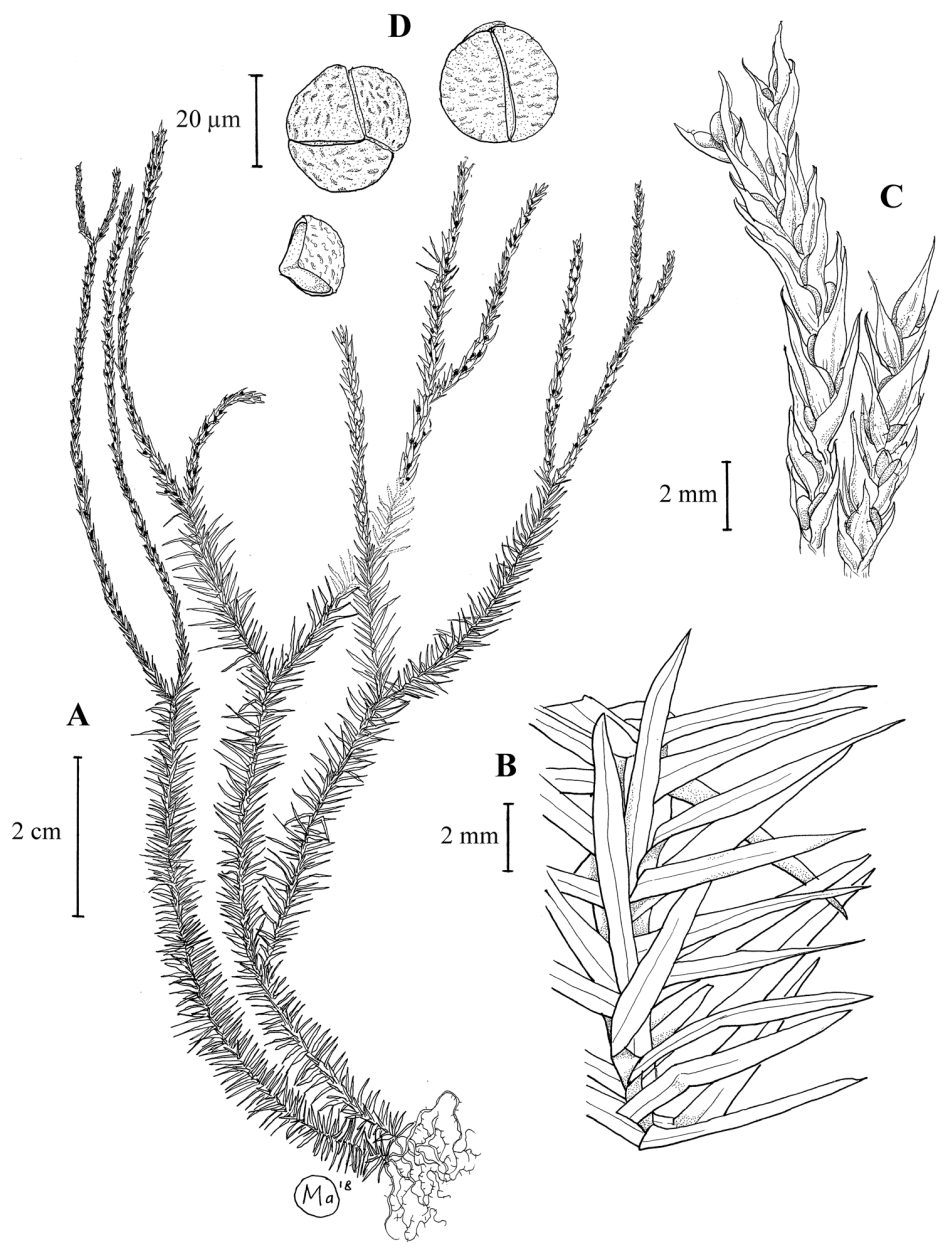
*Phlegmariurus monticola*. **A** habit **B** portion of leafy sterile branch **C** strobilus **D** spores. (Drawing by Mohamad Aidil Noordin from Ezzawanis et al. FRI 54517).

**Figure 4.**
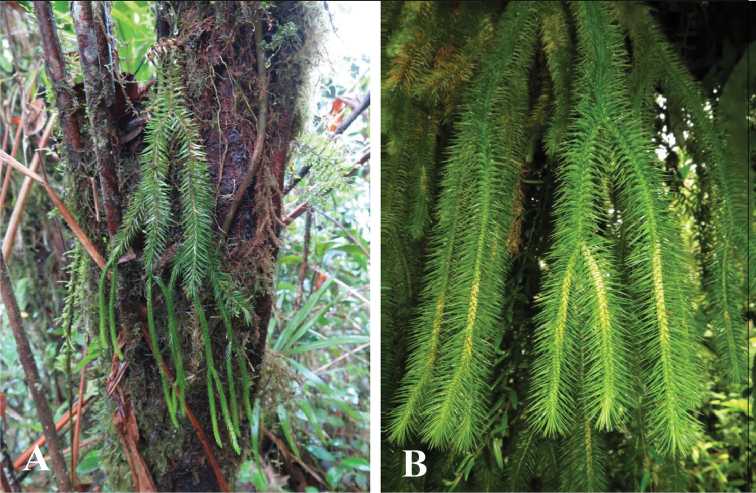
**A** *Phlegmariurus monticola* **B** Peninsular Malaysian form of *P. sqaurrosus* *s.l.* (Photographs Imin Kamin).

#### Diagnosis.

It belongs to the *Phlegmariurus squarrosus* group in that its sporophylls are similar in shape, although smaller, than the leaves. *Phlegmariurus monticola* is immediately distinct from *P. squarrosus* (G.Forst.) Á.Löve & D.Löve *s.l.* that in Peninsular Malaysia is morphologically relatively uniform by a combination of its tufted, shorter stems 11–32.5 cm long (vs. single-stemmed at the base and (20–)40–55(–200) cm long), leaf midrib obscure above and prominent beneath (not distinct above and faint beneath), strobili more slender ca. 3 mm wide, that are in pairs and usually branch dichotomously once or twice (vs. strobili 4–5 mm wide, single and always unbranched). In addition, their distributions do not overlap; *P. squarrosus* is a lowland species growing on trees usually at less than 300 m elevation, while *P. monticola* is a montane species occurring at 1400–2100 m elevation. It also superficially resembles *P. prolifera* (Blume) A.R Field & Bostok in its slender strobili ca. 3 mm wide and sporophylls arranged in four rows, but it differs in its shorter, narrower leaves (8–10 × 0.75–1.5 mm (vs. leaves 10–15 × 1.5–2 mm) and its narrowly lanceolate sporophylls 0.5-1 mm wide (vs. triangular-ovate sporophylls 1.5-2 mm wide).

#### Distribution.

Peninsular Malaysia (Kelantan, Perak and Pahang).

#### Provisional conservation status.

Least Concern. It is found in most accessible montane areas in the Main Range suggesting that it is likely to be more widespread. The montane forest above 1000 m is protected because of the restriction on clearing forest on steep slopes. However, this does not apply to hill resorts where forest is cleared for resort infrastructure and at Cameron Highlands for vegetable and flower farms (Kiew 1997). However, its populations need to be monitored because it is for sale in nurseries in Malaysia, Singapore and Thailand (AR Field, pers. comm.) so collecting of plants from the wild may become a threat.

#### Ecology.

In Peninsular Malaysia, in light shade usually in lower montane forest, sometimes in upper montane forest, at 1400–2100 m elevation.

#### Etymology.

Latin, *monticola* - dweller in mountains.

#### Notes.

In appearance, it is immediately distinct from *P. squarrosus* in being less robust, in being tufted with up to 7 stems, having shorter stems that are only about twice the length of the strobili and leaves with the midrib obscure above and prominent beneath; while Peninsular Malaysian individuals of *P. squarrosus* have single stems (not tufted) that are longer so their strobili are about a fifth or less the length of the stem and the leaf midrib is distinct above and faint beneath.

#### Specimens examined.

Kelantan: Gunung Chamar Imin et al. FRI 71786 (KEP, L); Sungai Kenerong Imin et al. FRI 68171 (KEP), Kueh et al. FRI 58410 (KEP). Pahang: Cameron Highlands Aishah 15 (KLU); Holttum SFN 23443 (SING), Holttum s.n. May 1936 (SING); Imin et al. FRI 68482 (KEP), Imin et al. FRI 71946 (KEP), FRI 74765 (KEP, L), Imin et al. FRI 87114 (KEP), Nor-Ezzawanis et al. FRI 54517 (KEP), Poore 1019 (KLU); Fraser’s Hill Henderson SFN 11507 (SING); Genting Highlands Lim et al. GHC 1 (KLU); Aishah 22 (KLU), Stone 15422 (KLU), Tan et al. FRI 77645 (KEP, TAIF). Perak: Birch’s Hill Burkill SFN 12739 (SING); Gunung Hijau Julius et al. FRI 53305 (KEP), Sinclair & Kiah SFN 38728 (SING).

##### New combination

*Lycopodium pinifolium* Blume (1828: 264) was described and recorded to occur from Malaya to Papua, but it was not until 1984 that this species appeared in Malaysian publications (as *Huperzia pinifolia* Trevis.) when it was recorded from the Gunung Mulu National Park, Sarawak (Parris et al. 1984) where it was keyed out with *Huperzia phlegmaria* and distinguished from that species by its linear-lanceolate leaves at least six times longer than broad with a cuneate base and by its sporophylls with acuminate tips that protrude beyond the sporangia. A detailed account described and illustrated this species (Johns 1991) based on specimens from Gunung Mulu grown in the Royal Botanic Gardens Kew. However, it was not until Parris and Latiff (1997) listed it that it appeared in Peninsular Malaysian publications. However, its identity became confused when subsequent publications illustrated different species under this name. For example, that of Aziz-Bidin (2002, figs. 10, 11) illustrated *P. tetrastichus* and that of Noraini et al. (2010, figs. 47, 48) figured *P. squarrosus*. Names on herbaria specimens were similarly in a muddle. A full description is therefore provided below.

The name *Lycopodium pinifolium* Blume was an illegitimate name because it had earlier been used for an African species by Kaulfuss (1824). In 1874, Trevisan described *Huperzia pinifolia* Trevis. as a new name: in fact he was conscious of the illegitimacy of the name of Blume because of the (older) name of Kaulfuss. In this case (International Code of Nomenclature, art. 7.4 (ICN 2012)) the new name is typified by the type of the replaced synonym. Blume (1828), in describing his species, recognised varieties B, C and D but for varieties C and D he noted ‘(an species?)’ implying that there was doubt that they belonged to this species. Specimens representing these varieties were all annotated by his hand, the labels noting only ‘Java’ without recording a collector, locality or date. The specimen annotated as var. C (barcode L 0057377) is closely similar to *P. proliferus* (Blume) A.R.Field & Bostock and does not belong to *P. pinifolius* and the specimen representing var. D is a piece too small to identify with certainty. The other two specimens both belong to *P. pinifolius* in their habit (relatively short dichotomously branching stems with dense, narrowly lanceolate leaves perpendicular to the stem) and the abruptly distinct, straight strobili. The typical variety (barcode L 0057375) has strobili that are branched dichotomously while var. B (barcode L0057376) has unbranched strobili, which represent an earlier stage in the growth of the strobili, which eventually branch. The herbarium sheet with barcode L 0057375 is here selected as the lectotype because it best represents a mature plant of this species.

### Phlegmariurus pinifolius

Taxon classificationPlantaeLycopodialesLycopodiaceae

(Trevis.) Kiew comb. nov.

urn:lsid:ipni.org:names:77177913-1

#### Basionym.

*Huperzia pinifolia* Trevis., Atti Soc. Ital. Sci. Nat. 17: 247. 1874 ≡ *Lycopodium pinifolium* Blume, Enum. Pl. Javae. 2: 264 (1828), non Kaulf. (1824).

#### Type.

Java. Without collector, number, date or precise locality (lectotype here selected: L!, electronic image with barcode L 0057375).

#### Description.

Medium-sized epiphyte, tufted with 2–4(–6) stems. **Stems** pendulous, 10–20(–50) cm long, ca. 1.5 mm diam., branching dichotomously 2–3(–4) times, branches equal. **Leaves** crowded, at right angles to stem, sessile, glossy, mid-green, thinly coriaceous, narrowly lanceolate, 6–8 × 1–1.7 mm, slightly narrowed at base, margin entire, slightly revolute, apex narrowed to a sharp point, glabrous, midrib obscure above, faint beneath. **Sporophylls** smaller, dissimilar to leaves, transition to strobilus abrupt. **Strobili** light green, 2–9 cm long, slender, 1–1.5 mm diam., branched at base to form a pair of straight branches, sometimes further branched once or twice. Sporophylls crowded, not arranged in 4 rows, sessile, green, base broadly ovate and partially covering the sporangium, narrowed abruptly above the sporangium to a short point, ca. 1.5 × 0.7 mm, margin entire, keeled outside. **Sporangium** discoid, ca. 1 mm diam.

#### Distribution.

Thailand, Peninsular Malaysia, Java, Borneo (Sabah and Sarawak), the Philippines, Indonesia (Sumatra to New Guinea). Hassler (2018) also records it from Sri Lanka, Vanuatu and the Solomon Islands. In Peninsular Malaysia, collected from Penang, Perak, Kelantan and Pahang.

#### Provisional conservation status.

Least Concern.

#### Ecology.

Not common, in upper hill dipterocarp to lower montane forest, in light shade, on trees on hill sides at 800–1250 m altitude.

#### Etymology.

Latin, -*folius* = leaf; referring to the similarity to leaves of *Pinus*.

## Supplementary Material

XML Treatment for Phlegmariurus iminii

XML Treatment for Phlegmariurus monticola

XML Treatment for Phlegmariurus pinifolius
